# Tumor suppressor pathways shape EGFR-driven lung tumor progression and response to treatment

**DOI:** 10.1080/23723556.2021.1994328

**Published:** 2022-01-14

**Authors:** Giorgia Foggetti, Chuan Li, Hongchen Cai, Dmitri A. Petrov, Monte M. Winslow, Katerina Politi

**Affiliations:** a Department of Internal Medicine (Medical Oncology), Yale Cancer Center, Yale School of Medicine, New Haven, Connecticut, USA; b Department of Medical Oncology, IRCCS Ospedale San Raffaele, Milan, Italy; c Departments of Biology, Stanford University School of Medicine, Stanford, California, USA; d Department of Genetics, Stanford University School of Medicine, Stanford, California, USA; e Department of Pathology, Stanford University School of Medicine, Stanford, California, USA; f Stanford Cancer Institute, Stanford University School of Medicine, Stanford, California, USA; g Department of Pathology, Yale School of Medicine, New Haven, Connecticut, USA; h Yale Cancer Center, Yale School of Medicine, New Haven, Connecticut, USA

**Keywords:** Lung cancer, EGFR, targeted therapy, tumor suppressor genes, multiplexed *in vivo* genome editing

## Abstract

*In vivo* modeling combined with CRISPR/Cas9-mediated somatic genome editing has contributed to elucidating the functional importance of specific genetic alterations in human tumors. Our recent work uncovered tumor suppressor pathways that affect EGFR-driven lung tumor growth and sensitivity to tyrosine kinase inhibitors and reflect the mutational landscape and treatment outcomes in the human disease.

Tyrosine kinase inhibitors (TKIs) are the standard of care treatment for oncogenic epidermal growth factor receptor (EGFR)-driven lung adenocarcinomas.^1^ Despite the efficacy of TKIs, responses are heterogeneous and drug resistance inevitably emerges, underscoring the need to identify determinants of therapeutic sensitivity.^1^ Understanding how genotypes influence responses to drugs could help advance treatment strategies for different subsets of patients with EGFR-driven lung cancer and delay or prevent the emergence of resistance. Sequencing data of human tumors show that *EGFR* alterations co-occur with alterations in many putative tumor suppressor genes.^2^ Whether these alterations have biological implications and whether their relative alteration frequency reflects their functional importance remains largely unknown. Understanding the extent to which tumor suppressor gene alterations contribute to TKI sensitivity and resistance could improve treatment approaches (Figure 1). Moreover, the identification of combinations of genetic alterations that alter tumor fitness could also lead to the discovery of novel vulnerabilities of genomic subsets of *EGFR* mutant tumors.^2^

**Figure 1.**
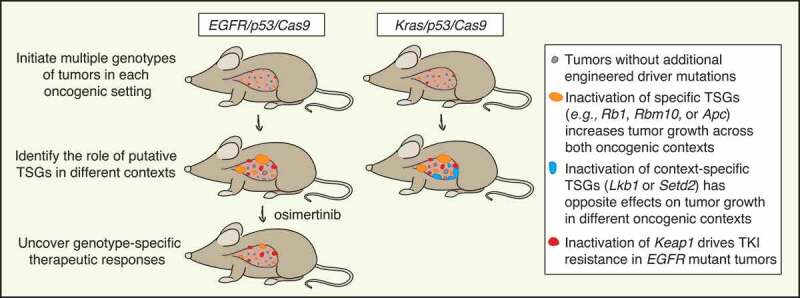
Dissecting the role of tumor suppressor genes using multiplexed genome editing across oncogenic contexts. Schematic of multiplexed genome editing in a mouse model of *Epidermal growth factor receptor* with activating point mutation L858R (*EGFR^L858R^*) mutant and *Transformation related protein 53* (*Trp53*, best known as *p53*)-deficient lung adenocarcinoma (expressing Cas9; *EGFR/p53/Cas9*). Tumors are initiated by intratracheal administration of a lentiviral pool of vectors that lead to simultaneous tumor suppressor gene (TSG) inactivation mediated by CRISPR/Cas9 system. We modeled *EGFR/p53* mutant tumors and 10 tumor genotypes via inactivation of putative tumor suppressor genes and quantified their impact on tumor growth compared to a different oncogenic context (*Kirsten rat sarcoma viral oncogene homologue* with activating point mutation G12D, *Kras^G12D^* mutant and *p53*-deficient model; *Kras/p53/Cas9*) and on sensitivity to treatment with osimertinib.

Prior to our study, there were almost no *in vivo* studies on the function of tumor suppressor genes in oncogenic EGFR-driven lung cancer. This was at least partially due to the absence of suitable autochthonous EGFR-driven lung cancer models with which to investigate the biological consequences of gene inactivation.^3^ In our recent work, we leveraged tumor barcoding with high-throughput barcode sequencing (Tuba-seq)^4, 5^ and applied it to a novel model of *EGFR* mutant and *Transformation related protein 53* (*Trp53*, best known as *p53*)-deficient lung adenocarcinoma to study the functional importance of tumor suppressor genes that are frequently altered in human lung tumors.^3 –6^ Our findings revealed tumor suppressor genes that when inactivated promoted tumor growth, most notably *RNA binding motif protein 10* (*Rbm10), Retinoblastoma 1* (*Rb1*), and *Adenomatous polyposis coli* (*Apc*).^3^ Importantly, these tumor suppressor genes were also some of the most frequently altered genes in *EGFR/P53* mutant human lung tumors, supporting the importance of these tumor suppressor pathways in driving *EGFR* mutant lung tumors.^3^ *AT-rich interaction domain 1A* (*Arid1a*) and *Cyclin-dependent kinase inhibitor 2A* (*Cdkn2a*) inactivation appeared to increase tumor growth only later during tumor progression, suggesting that specific tumor suppressor genes can have different effects at different stages of tumor development. Inactivation of other putative tumor suppressor genes that we investigated did not promote tumor growth, indicating that the function of some tumor suppressor genes may be highly context-dependent (Figure 1).^3^

Mutual exclusivity between genomic alterations in human tumors can imply redundancy of biological processes or synthetic lethality. Given the complexity of tumorigenesis, multiple factors (*e.g*., tumor subtype, mutational processes and load, environment) may play a role in determining genetic epistasis, suggesting that experimental approaches are particularly important for understanding why this is observed.^7^ Oncogenic *EGFR* alterations (mainly affecting *EGFR* exons 18 through 21) and oncogenic *Kirsten rat sarcoma viral oncogene homologue* (*KRAS*) missense mutations (mainly at codons 12, 13, and 61) represent major drivers of lung adenocarcinoma.^2^ Given that both these oncogenes are components of the same pathway, we anticipated that tumor suppressor genes would have similar impacts on *in vivo* growth of *EGFR* and *KRAS* mutant lung tumors. Although inactivation of *Rbm10, Rb1*, or *Apc* did have similar effects on *EGFR* and *KRAS* mutant tumor growth, *Serine/threonine kinase 11* (*Stk11*, also known as *Lkb1*) and *SET domain containing 2, histone lysine methyltransferase* (*Setd2*) inactivation had opposite effects in the two oncogenic settings. *Lkb1* and *Setd2* inactivation are two of the strongest drivers of *KRAS* mutant tumor growth; however, their inactivation reduced *EGFR* mutant tumor growth.^8^ These results correlate with the relative frequency of *LKB1* and *SETD2* alterations in human *EGFR* and *KRAS* mutant lung tumors, suggesting the existence of a synthetic lethal relationship between *Lkb1*/*Setd2* inactivation and oncogenic *EGFR*. These findings underscore the importance of quantitative modeling of genetic alterations *in vivo*. More broadly, the observation that inactivation of certain genes can have different effects depending on the specific oncogenic alteration present (*e.g., KRAS* vs. *EGFR* mutation) revealed surprising context specificity of the role of these genes in cancer (Figure 1).^3^

Osimertinib, a third generation TKI that leads to better overall survival compared to other TKIs, has been approved as first-line therapy for patients with metastatic EGFR-driven lung cancer.^9^ Recently, osimertinib was also approved as adjuvant therapy for early-stage *EGFR* mutant tumors.^10^ Outcomes upon osimertinib treatment are variable; thus, the importance of uncovering how co-incident genomic alterations contribute to sensitivity and resistance is of great clinical relevance. We quantified the impact of inactivating 10 putative tumor suppressor genes on the response to osimertinib within our mouse model and found that *Kelch-like ECH associated protein 1* (*Keap1*) inactivation reduced sensitivity to this therapy.^3^ Our *in vivo* data mirror clinical data suggesting that KEAP1 pathway alterations predict poor clinical responses to TKIs.^3^ Thus, we established a causal link between this tumor suppressor pathway and TKI sensitivity in *EGFR* mutant lung adenocarcinoma.

The multiplexed *in vivo* CRISPR/Cas9 screening approach that we used allows us to interrogate multiple genes simultaneously and assess their contributions to tumor phenotypes.^3,4,5,6, 8,11^ Thus, it provides quantitative cause-and-effect information and avoids confounding factors that are inevitable in human tumors (*e.g*., high mutation burden). However, our model systems do not yet recreate the extent of genomic complexity and intratumoral heterogeneity found in human lung tumors. Future studies will also be required to uncover the molecular mechanisms by which these tumor suppressors normally constrain tumor growth and by which their inactivation sensitizes tumors to therapy. Our study represents an initial step in defining the role of tumor suppressor genes in oncogenic EGFR-driven lung tumors. We anticipate that assessing broader panels of putative tumor suppressors will further elucidate the functional genomic landscape of this disease and allow genotypes to be related to diverse cancer phenotypes, including their response to different therapies. Despite the efficacy of single agent osimertinib, quantifying the impact of rational combination therapies on defined genotypes of lung tumors will drive further gains in the treatment of specific subsets of tumors. We envision that these types of multiplexed *in vivo* studies will ultimately contribute to the development of tailored treatments for patients with *EGFR* mutant lung cancer.
